# The role of extracorporeal membrane oxygenation in the management of rapidly progressive interstitial lung disease due to anti-melanoma differentiation-associated gene 5 antibody dermatomyositis: A case series and brief literature review

**DOI:** 10.1016/j.jhlto.2026.100518

**Published:** 2026-02-10

**Authors:** James M. Jurica, Mazen F. Odish, Christine M. Lin, Robert L. Owens, Cassia Yi, Michelle Parrett, Alisha Kabadi, Shannon LeBlanc, Chelsea Roche, Gordon Yung, Jisha Joshua, Soo-In Choi, Eugene Golts, Travis Pollema, Kamyar Afshar

**Affiliations:** a UC San Diego Department of Medicine, Division of Pulmonary, Critical Care, Sleep Medicine, and Physiology, San Diego, CA; b UC San Diego Department of Medicine, Division of Rheumatology, Autoimmunity and Inflammation, La Jolla, CA; c UC San Diego Department of Surgery, Division of Cardiovascular and Thoracic Surgery, La Jolla, CA

**Keywords:** ECMO, Rapidly progressive ILD, RP-ILD, MDA5 dermatomyositis, Lung transplantation, Physical therapy

## Abstract

**Introduction:**

Rapidly progressive interstitial lung disease (RP-ILD) from anti-melanoma differentiation-associated gene 5 antibody (anti-MDA5) dermatomyositis (DM) is a rare condition associated with up to 63% mortality. Despite aggressive medical treatment, many patients require escalating levels of respiratory support. Extracorporeal membrane oxygenation (ECMO) has emerged as a rescue therapy for these patients, intended as a bridge to lung transplantation.

**Methods:**

A retrospective chart review was conducted on patients who were treated at our center between 2021 and 2025. Baseline characteristics, treatments, complications, and outcomes are described and presented herein. A literature review was performed to compare our outcomes for patients with anti-MDA5 DM placed on ECMO with those reported elsewhere.

**Results:**

The study included 9 patients (5 females and 4 males) with RP-ILD due to anti-MDA5 DM. All patients were treated with high-dose corticosteroids and a variety of other immunosuppressive therapies. Eight patients were placed on veno-venous (V-V) ECMO and one patient was placed on veno-arterialvenous (V-AV) ECMO. All patients were evaluated for lung transplant candidacy. Eight of nine patients were deemed ineligible for lung transplant, most commonly due to severe deconditioning, and died in the ICU. One patient was successfully bridged to bilateral lung transplant after 3 days on ECMO and is currently 30 months post-transplant.

A retrospective review of published studies found 79 patients with anti-MDA5 DM induced-RP-ILD requiring ECMO support. Twenty-eight (35.4%) patients were bridged to transplant (average time on ECMO 18 days), 46 (58.2%) died in the ICU (average time on ECMO 28 days), and only 4 (5%) were successfully bridged to recovery (average time on ECMO 44 days). Outcomes for 1 patient were not reported.

**Conclusion:**

RP-ILD due to anti-MDA5 DM is associated with a high rate of mortality despite current medical therapies. ECMO as a bridge to recovery is very unlikely (∼5%, 4/79) for these patients; thus, lung transplant should be pursued expeditiously, particularly given the possible association between shorter time on ECMO and successful transplantation. Transfer to an ECMO and lung transplant center is crucial since immunosuppression decisions should be made in a multidisciplinary fashion (lung transplant, rheumatology, etc.) as these decisions can affect lung transplant candidacy.

## Background

Anti-melanoma differentiation-associated gene 5 antibody (anti-MDA5) dermatomyositis (DM) is an idiopathic inflammatory myopathy (IIM) and subtype of clinically amyopathic DM typically associated with rash, polyarthralgia, and increased risk for development of rapidly progressive interstitial lung disease (RP-ILD). The exact etiology of anti-MDA5 DM remains unknown, but it is believed to be due to complex pathomechanism involving the innate and acquired immune system. MDA5 is a cytosolic sensor for viral double-stranded RNA and viral infection is posited to trigger an autoimmune, hyperinflammatory response.^1^ The prevalence of anti-MDA5 DM varies by population and is estimated to comprise 1.3%-10% of patients with IIM in European patients, while in Asian cohorts the prevalence is believed to be between 15% and 36% of patients with IIM.^2^, ^3^ RP-ILD is the most feared complication and is frequent in this population, with reported rates of roughly 57%.^4^

Treatment for patients with RP-ILD due to anti-MDA5 DM is variable. Nearly all patients are treated with high-dose glucocorticoids. Many are treated with calcineurin inhibitors, cyclophosphamide, rituximab, or a range of other immunosuppressants in various combinations. More recently, Janus kinase inhibitors such as tofacitinib and baricitinib have been utilized as adjunctive therapies with promising results.^5^, ^6^ Other modalities such as IVIG and plasmapheresis are often utilized either with initial treatment or for patient’s refractory to other medical therapies.^7^ These patients face high mortality rates, as up to 79% of patients with RP-ILD have progressive disease despite therapy.^8^

For patients who experience worsening respiratory function despite medical therapy, extracorporeal membrane oxygenation (ECMO) has emerged as a rescue therapy intended to bridge patients to lung transplantation. However, little data exist with regards to complications, outcomes, and opportunities for optimization in this population. In this paper, we seek to add to the existing literature by presenting our experience of 9 patients with RP-ILD due to anti-MDA5 DM on ECMO treated at our institution between 2021 and 2025, to briefly review the existing literature with regards to treatment and outcomes, and to provide recommendations in this high-risk population.

## Materials and methods

### Case series chart review

This is a retrospective case series of adults (≥18 years) diagnosed with anti-MDA5 DM with RP-ILD who required ECMO at our center between December 2021 and August 2025. The diagnosis of anti-MDA5 DM was based on clinical and radiographic evidence and must include a positive anti-MDA5 antibody testing during admission. This study was approved by the Institutional Review Board at the University of California San Diego (IRB# 190181X).

All patient data (demographics, comorbidities/medical history, laboratory data, and disease course) were extracted from the electronic medical record at our institution related to individual cases. Initial laboratory data are reported only if obtained within 7 days of hospitalization. Disease duration is reported from initial symptoms related to underlying anti-MDA5 DM to hospitalization and is reported based on physician documentation. Time from admission to ICU was calculated based on hospitalization during which the patient required ECMO support. Follow-up duration is based on the last documentation in the electronic medical record.

### Brief literature review

A literature search of EMBASE and PUBMED/Medline was conducted in November 2025 using the following search concepts and Boolean operators: “Rapidly Progressive Interstitial Lung Disease” OR “RP-ILD” AND “Anti-melanoma differentiation associated gene 5″ OR “MDA5″ AND “extracorporeal membrane oxygenation” OR “ECMO.” No articles were added by contacting experts or other authors. All studies between 2005 and 2025 were included in the search. A total of 99 and 87 articles were identified in EMBASE and PUBMED/Medline, respectively. All duplicate articles were removed, and only studies in English were included. All abstracts and pediatric cases (<18 years old) were excluded. All articles describing cases of RP-ILD due to anti-MDA5 DM in which patients were not placed on ECMO were excluded. Articles examining ECMO in patients with RP-ILD due to causes other than anti-MDA5 DM were included only if they also contained patients noted to have received ECMO for RP-ILD due to anti-MDA5 DM. Data pertaining to patients with RP-ILD not due to anti-MDA5 DM from these studies were excluded as possible. These articles, and the patient cases presented therein who developed RP-ILD due to a disease process other than anti-MDA5 DM, are clearly identified in the paper and in Table 3. A total of 33 articles meeting the above criteria were included in the final literature review. A single reviewer performed the literature review and extracted data from each of the included studies. Extracted data included: total number of patients, age, sex, race, comorbidities, immunosuppressive regimen (prior to transplant), number of patients transplanted, number of patients recovered, number of patients died, days on ECMO to transplant, recovery, or death, and overall mortality.

### Statistical analysis

Continuous variables are expressed as a median with interquartile range. Categorical variables are expressed as a raw number with an associated percentage. Given only 1 patient received lung transplantation, no comparisons were made between this patient and the other patients in the case series.

## Results

All 9 patients included in our study were first hospitalized within 3 months of initial diagnosis. Fifty-six percent (5/9) of patients in this study were female, with 33% (3/9) Asian and 22% (2/9) white. The median duration of symptoms at time of hospitalization for RP-ILD was 76 [55-90] days. All 9 patients presented with skin findings (e.g., Heliotrope rash, Gottron Papules), 44% (4/9) presented with fever, and 56% (5/9) presented with myalgias, see Table 1 and Supplementary Table S1.

**Table 1:** Baseline Characteristics at Hospital Discharge

Baseline characteristics at hospital admission	Died in ICU (*n* = 8)	Survived to transplant (*n* = 1)
*Age [IQR]*	47 [39,50]	52
*Female (%)*	4 (50)	1
*Race/Ethnicity (%)*		
Asian	2 (25)	1
White	2 (25)	0
Black	1 (12)	0
Other	3(38)	0
BMI	30 [27,32]	26
Charlson Comorbidity Index [IQR]	2 [1,2]	2
Approximate duration in days of symptoms at time of hospitalization [IQR]	68 [55,90]	120
*Signs and symptoms at presentation (%)*		
Fever	4 (50)	0
Gottron papules	6 (75)	1
Heliotrope rash	6 (75)	0
Mechanic hands	1 (13)	0
Articular symptoms	5 (63)	0
Muscular symptoms	5 (63)	0
*Lab markers*		
Anti-MDA5 (%)	8 (100)	1
CPK (U/L) [IQR]	73 [59,96]	N/A
LDH (U/L) [IQR]	431 [298,590]	N/A
Ferritin (ng/mL) [IQR]	1425 [770,2243]	N/A
CRP (mg/dL) [IQR]	33 [3,38]	0.36
Anti-SSA positivity (%)	1 (13)	1
Anti-synthetase antibody positivity (%)	0	0
Anti-smith positivity (%)	1 (13)	0
dsDNA positivity (%)	0	N/A
AST (U/L) [IQR]	69 [60,191]	28
ALT (U/L) [IQR]	80 [57,186]	55
*First CT chest post-diagnosis (%)*		
OP	4 (50)	1
NSIP	2 (25)	0
DAD	0	0
UIP	0	0
Indeterminate	2 (25)	0

Abbreviations: ALT, alanine aminotransferase; AST, aspartate aminotransferase; BMI, body mass index; CPK, Creatine phosphokinase; CRP, C-reactive protein; DAD, diffuse alveolar damage; dsDNA, double stranded DNA; NSIP, non-specific interstitial pneumonia; OP, organizing pneumonia; UIP, usual interstitial pneumonia.

Labs within 7 days of hospital admission. Median value is reported for all continuous variables unless otherwise specified.

One (1/9, 11%) patient had an elevated serum CPK at diagnosis. Four (44%) patients had elevated ferritin on admission, with a median level of 1425 ng/mL [770-2243]. Median CRP was also highly elevated at 19 mg/dL [IQR 3,38]. Two patients had positive anti-SSA, 1 patient had a positive anti-smith, and no patients had an elevated anti-synthetase antibody. On initial CT chest after diagnosis, 56% (5/9) of patients were found to have organizing pneumonia, 22% (2/9) were noted to have nonspecific interstitial pneumonia, no patients had usual interstitial pneumonia, and 22% (2/9) were considered indeterminate usual interstitial pneumonia.

All patients received immunosuppression prior to initiation of ECMO. All 9 patients received pulse-dose steroids (500-1000 mg of methylprednisolone per day for 3 days) during hospitalization. Fifty-six percent (5/9) of patients received rituximab or cyclophosphamide, 33% (3/9) received hydroxychloroquine, 22% (2/9) received mycophenolate or azathioprine, and 1 patient (13%) received anakinra, see Table 2 and Supplementary Table S1. Eighty-nine percent (8/9) of patients were initiated on veno-venous (V-V) ECMO, with 1 patient on veno-arterialvenous (V-AV) ECMO. The median time from ICU admission to ECMO initiation was 6 days [IQR 2,13].

**Table 2:** Clinical Course and Outcomes

Clinical course and outcomes	Died in ICU (*n* = 8)	Survived to transplant (*n* = 1)
*Hospital admission*		
SOFA at time to hospitalization [IQR]	6 [5,7]	N/A
*Treatments prior to ICU admission (%)*		
Pulse dose steroids	4 (50)	0
Rituximab	3 (38)	0
Hydroxychloroquine	3 (38)	0
Azathioprine	1 (13)	0
Mycophenolate	2 (25)	0
Plasma exchange	0	0
IVIG	3 (38)	0
Days from hospitalization to ICU	5 [3,15]	0
*ICU Admission*		
Time from ICU to intubation (days)	0 [0,0]	10
SOFA at time of ICU admission [IQR]	6 [6,9]	N/A
*Treatments in the ICU (%)*		
Pulse dose steroids	4 (50)	1
Rituximab	2 (25)	1
Cyclophosphamide	5 (63)	0
Tacrolimus	4 (50)	1
Mycophenolate	1 (13)	0
Hydroxychloroquine	1 (13)	0
Anakinra	1 (13)	0
Plasma exchange	3 (38)	0
IVIG	5 (63)	1
*Complications in ICU (%)*		
Sepsis	6 (75)	1
Disseminated intravascular coagulation	1 (13)	0
Continuous renal replacement therapy	6 (75)	0
Acute blood loss	5 (63)	0
Hemolysis	3 (38)	0
Pneumothorax/Pneumomediastinum	6 (75)	0
Digital ischemia	3 (38)	0
Heart failure	5 (63)	0
*ECMO parameters*		
SOFA at time of ECMO cannulation [IQR]	6 [6,9]	8
V-V ECMO (%)	7 (88)	1
V-AV ECMO (%)	1 (13)	0
Time from ICU to ECMO (days) [IQR]	5 [2,13]	3
Time from ECMO to death (days) [IQR]	30 [18,53]	N/A
Time from ECMO to transplant (days)	N/A	3
*Best participation with physical therapy (%)*		
No participation with physical therapy	3 (38)	0
Sitting edge of bed	3 (38)	0
Sit to stand	1 (13)	1
Walking	1 (13)	0
*Cause of death (%)*		
Deconditioning No longer transplant candidate Transitioned to comfort care measures only	4 (50)	N/A
Septic shock	2 (25)	N/A
Other	2 (25)	N/A

Median value is reported for all continuous variables unless otherwise specified.

IVIG, Intravenous Immunoglobulin; SOFA, Sequential Organ Failure Assessment; V-V ECMO, veno-venous extracorporeal membrane oxygenation; V-AV ECMO, veno-arterialvenous extracorporeal membrane oxygenation.

The patients' complications and outcomes can be seen in Table 2. For 56% (5/9) of patients, the ICU course was complicated by bacterial pneumonia. Sixty-seven percent (6/9) of patients developed either pneumothorax or pneumomediastinum during hospitalization. Fifty-six percent (5/9) of patients required continuous renal replacement therapy at some point during hospitalization, and 56% (5/9) of patients developed either right or left ventricular dysfunction while in the ICU. With regards to ECMO-specific complications, 33% (3/9) of patients required transfusion for hemolysis (presumed secondary to the ECMO circuit), 1 patient had a clinically significant hemorrhage during cannulation, and 1 patient was disconnected from oxygen to the membrane oxygenator, resulting in a brief cardiac arrest with return of spontaneous circulation. With regards to physical conditioning, only 1 patient (13%) was strong enough to walk while on ECMO, 1 (13%) was able to perform sit to stands, 3 (33%) were able to sit at the edge of the bed, and 3 (33%) were unable to work with physical therapy due to the severity of illness and myopathy.

Overall, 89% (8/9) of patients died in the ICU with 1 patient bridged to transplant, and no patients successfully bridged to recovery. The time between ECMO initiation and transplant was 3 days for the patient who survived. The median time from ECMO initiation to death in the other 8 patients was 30 [18,53] days. The cause of death for 50% (4/8) was withdrawal of care in the setting of severe deconditioning, making the patient no longer a transplant candidate, making this the most common cause of death. Twenty-five percent of patients (2/8) died from septic shock, and another 25% (2/8) of patients died from progressive multiorgan failure with no clear inciting cause.

### Brief literature review

A retrospective review of 33 published studies identified 79 patients with anti-MDA5 DM-induced RP-ILD who required ECMO support, see Table 3. The median age of these patients was 51 years old. Essentially all patients received glucocorticoids, with nearly all patients receiving other immunosuppressants, including cyclophosphamide, tacrolimus, IVIG, plasmapheresis, rituximab, etc. Of the 79 patients identified in the literature, 28 (35.4%) underwent lung transplantation, 46 (58.2%) died, and only 4 (5%) patients were bridged to recovery. While time from ECMO to transplant or death was variably reported in the literature (56/79, 71%), for those studies in which this timeframe was available, average time from ECMO to transplant was notably shorter than time from ECMO to death (18 days vs 28 days).

**Table 3:** Literature Review of Published Cases of ECMO for RP-ILD Due to Anti-MDA5 DM

Study	Total patients with anti-MDA5 DM	Age (Median)	Female (%)	Race (%)	Comorbidies (%)	Immunosuppression (%)	Transplanted	Bridged to recovery	Died	Days on ECMO to transplant	Days on ECMO to recovery	Days on ECMO to death	Mortality (%)
Zheng et al (2025)^9^	15	47	50*	Asian - 14, Black - 5, Caucasian - 76, Other - 5*	Charlson comorbidity index 2 [2, 2] (IQR)*	GC - 100, CYC - 73, IVIG - 46, MMF - 36, RTX - 36, CNI - 32, AZA - 5, PLEX - 23*	6	2	7	30 [15-41] Median(IQR)*	11 [7,27] Median(IQR)*	24 [18,37] Median(IQR)*	53
Bay et al (2022)^10^	15	50	80	Unknown	N/A	GC - 87, CYC - 47, CNI - 33, TOF - 13, MTX - 7, MMF - 13, RTX - 13**	5	0	10	8 (4-20) (Range)	N/A	30 (8-52) (Range)	66
Wang et al (2024)^11^	7	58	43	Unknown	T2DM - 14	GC - 100, JAKi - 100, IVIG - 100, CYC - 14, CNI - 43)	0	0	7	N/A	N/A	11 (6-12) (Range)	100
Rubin et al (2021)^12^	6	52*	56*	Black - 22, Hispanic 11, White - 67*	Obesity - 22, TUD - 22, AUD - 22, GERD - 22, HTN - 33*	GC - 100, RTX - 78, IVIG - 78, MMF - 11, CYC - 22*	0	1	5	N/A	52	41 (mean)	83
Jade et al (2025)^13^	5	47	60	Asian - 60, White - 20, Other - 20*	Wilson's Disease - 20, NAFLD - 20, Psoriasis - 20, Eczema - 20, T2DM - 20, latent TB - 20	GC - 100, CYC - 60, IVIG 60, RTX - 80, MTX - 20, CsA - 20, MMF - 20, Tac - 20, AZA - 20, HCQ - 20	5	0	0	Unknown	N/A	N/A	0
Biddle et al (2024)^14^	2	54	100	Asian - 50, Other - 50	None	GC - 100, TOF - 100, Tac - 100, CYC - 100, RTX - 100	0	0	2	N/A	N/A	42 (mean)	100
Ismail et al (2025)^15^	2	53	50	Black - 50, Hispanic - 50	Obesity - 50, OSA - 50, CAD - 50, HTN - 50, HLD - 50, TUD - 50, Asthma - 50, Thymectomy - 50, SLE - 50	GC - 100, CYC - 50, Tac - 50, RTX - 50, IVIG - 100	0	0	2	N/A	N/A	53 (mean)	100
Lian et al (2023)^16^	2	62	0	Asian - 100	TUD - 50	GC - 100, CYC - 100	2	0	0	8 (Mean)	N/A	N/A	0
Hage et al (2025)^17^	1	51	0	Unknown	None	GC, CYC, CsA, PLEX	1	0	0	Unknown	N/A	N/A	0
Zhang et al (2025)^18^	1	59	0	Unknown	T2DM, HTN	GC, IVIG, Tac, Tac, TOC, TOF	1	0	0	6	N/A	N/A	0
Quy et al (2025)^19^	1	66	0	Unknown	TUD	GC, Tac	0	0	1	N/A	N/A	Unknown	100
Roane et al (2024)^20^	1	43	0	Unknown	Unknown	GC, IVIG	1	0	0	Unknown	N/A	N/A	0
Subramanian et al (2024)^21^	1	52	100	Unknown	Asthma	GC, IVIG, CYC, Tac	0	0	1	N/A	N/A	Unknown	100
Leveque et al (2023)^22^	1	24	100	African	IDA	GC, PLEX, Tac, TOF	1	0	0	4	N/A	N/A	0
Onose et al (2023)^23^	1	53	0	Unknown	Lupus Profundus, TUD	GC, CYC, Tac	0	0	1	N/A	N/A	17	100
Kenyon et al (2023)^24^	1	37	0	Caucasian	None	GC, CYC, PLEX, IVIG	0	0	1	N/A	N/A	Unknown	100
Biddle et al (2023)^25^	1	60	100	Asian	None	Unknown	0	0	1	N/A	N/A	Unknown	100
Zhang et al (2023)^49^	1	59	0	Unknown	T2DM, HTN	GC, IVIG, Tac, TOC, TOF	1	0	0	6	N/A	N/A	0
Ostendorf et al (2023)^26^	1	55	100	Unknown	None	GC, IVIG, CYC, TOF, CsA, Daratumumab	0	1	0	N/A	∼100	N/A	0
Thompson et al 2023^27^	1	56	0	Unknown	Unknown	PLEX*	0	0	1	N/A	N/A	Unknown	100
Al-Husayni et al (2022)^28^	1	46	0	Unknown	None	GC, CYC	0	0	1	N/A	N/A	Unknown	100
Esteva et al (2022)^29^	1	45	0	Black	T1DM, HTN, HLD	GC	0	0	1	N/A	N/A	Unknown	100
Anderle et al (2022)^30^	1	20	0	Asian	None	GC, CYC, Tac	1	0	0	31*	N/A	N/A	0
Tamaki et al (2022)^31^	1	32	100	Unknown	AML, Allogeneic-HCT, Graft vs. Host Disease	GC, CsA, CYC	0	0	1	N/A	N/A	Unknown	100
Borio et al (2021)^32^	1	62	0	Unknown	Hypertension	GC, IVIG	N/A	N/A	N/A	Unknown	N/A	N/A	N/A
Gu et al (2021)^33^	1	36	100	Unknown	None	GC	1	0	0	31	N/A	N/A	0
Marchiset et al (2021)^34^	1	44	100	Unknown	Unknown	GC, CYC, PLEX, TOF, Tac	1	0	0	20	N/A	N/A	0
Li et al (2020)^35^	1	27	100	Hispanic	Hirsutism	GC, IVIG, RTX	0	0	1	N/A	N/A	26	100
Pacot et al (2020)^36^	1	51	0	Unknown	TUD, HLD	GC, CYC, CsA, PLEX	1	0	0	Unknown	N/A	N/A	0
Manglani et al (2020)^48^	1	37	100	Hispanic	HTN, HLD	GC	0	0	1	N/A	N/A	Unknown	100
Aoyama et al (2019)^37^	1	47	0	Asian	Asthma	GC, CYC, Tac	0	0	1	N/A	N/A	38	100
Alqatari et al (2018)^38^	1	49	100	Unknown	None	GC, RTX, IVIG, CYC, Tac	0	0	1	N/A	N/A	unknown	100
Deitchman et al (2019)^39^	1	51	0	Caucasian	None	GC, MMF	1	0	0	<16 days	N/A	N/A	0
*Total (33 Articles)*	79	51 (Median)	53 (Average)	N/A	N/A	N/A	28	4	46	18 (Average)	44 (Average)	28 (Average)	60 (Average)

Information designated with an * contains information from both anti-MDA5 DM patients and non-anti-MDA5 DM patients.

AZA, azathioprine; AUD, alcohol use disorder; CT, computed tomography; CAD, Coronary Artery Disease; CMV, Cytomegalovirus; CNI, Calcineurin inhibitor; CYC, cyclophosphamide; DAD, diffuse alveolar damage; DAH, diffuse alveolar hemorrhage; GC, glucocorticoids; GERD, gastroesophageal reflux disease; HCQ, hydroxychloroquine; HCT, hematopoietic stem cell transplantation; HLD, hyperlipidemia; HTN, hypertension; HZV, Herpes Zoster; IDA, Iron Deficiency Anemia; IVIG, intravenous immunoglobulins; MDD, major depressive disorder; MMF, mycophenolate mofetil; MP, Methylprednisolone; MSSA, Methicillin-Susceptible Staphylococcus aureus; MRSA, Methicillin-Resistant Staphylococcus aureus; OSA, Obstructive Sleep Apnea; PJP, Pneumocystis jirovecii pneumonia; PLEX, Plasmapheresis; PNA, Pneumonia; RTX, rituximab; RV, Right ventricle; T1DM, type 1 diabetes mellitus; T2DM, type 2 diabetes mellitus; TAC, tacrolimus; TOC, Tocilizumab; TOF, tofacitinib; TUD, tobacco use disorder.

## Discussion

Our single-center retrospective case series further illustrates the high mortality associated with RP-ILD in patients with anti-MDA5 DM requiring ECMO support. The mortality rate seen in our study (89%) is higher than previously reported in the literature. This could be for multiple reasons. First, possible delayed presentation to the transplant center. All the patients at our center were initially hospitalized at outside institutions with multiple patients transferred on ECMO support for transplant evaluation after failure of medical therapy. Given these patients had disease that was refractory to medical therapy, they may represent a sicker cohort than found in other similar case reports/series. Additionally, similar case series have often included patients with RP-ILD due to other IIMs such as anti-synthetase syndrome, which is associated with a better prognosis and higher likelihood of bridging to recovery.^40^, ^41^ Finally, institutional variation in lung transplantation eligibility criteria may play a substantial role in overall survival of these patients given the extremely low likelihood of lung recovery.

With regards to utilization of ECMO as a bridge to recovery for patients with RP-ILD due to anti-MDA5 DM, our experience is consistent with previous studies, which have noted very little success (5%, 4/79). Despite a median time on ECMO of 30 days, none of the patients in our study showed improvement in lung function to the point that ECMO decannulation was ever considered. In contrast, for those patients who are appropriate candidates, ECMO as a bridge to transplant offers up to 60% survival at 1 year.^41^, ^42^, ^43^ As transplant gives the best chance of survival for these patients, expeditious transfer to a lung transplant and ECMO center should be pursued as early as the time of initial hospital admission for the following reasons: 1. Certain immunosuppressive regimens used to treat anti-MDA5 DM may affect transplant candidacy; thus, a multidisciplinary discussion between rheumatology and lung transplant physicians is essential to ensuring an appropriate immunosuppressive regimen is chosen. While the vast majority of commonly used immunosuppressive medications/regimens are not contraindicated prior to transplant at our center (apart from long-term high-dose steroids due to poor wound healing), certain regimens predispose patients to complications that negatively affect transplant candidacy, and each patient should be evaluated on an individual basis. Examples of complications seen in our cohort include acute blood loss following plasmapheresis and infections following cyclophosphamide. Infectious complications may be decreased with other regimens such as Rituximab, further emphasizing the need for multidisciplinary discussions between rheumatology and lung transplant teams. The heterogeneity of immunosuppressive regimens seen in our cohort is largely because all patients were initially hospitalized and treated at outside institutions prior to transfer and because there are minimal data comparing the efficacy of different immunosuppressive regimens in patients already on ECMO. 2. Response to initial treatment should be monitored closely, as patients with refractory disease (e.g., those who progress to requiring ECMO) will likely benefit from deescalation of immunosuppression once the decision is made to use ECMO as a bridge to lung transplant. Given that secondary infections occurred in 78% of patients in our series, de-escalation of immunosuppression in lung transplant candidates may help reduce this risk. For this reason, multiple patients in our cohort had their immunosuppression reduced to moderate-dose prednisone (10-30 mg/day) alone after initiation of ECMO. 3. Transferring to an appropriate lung transplant and ECMO center may allow for assessment of transplant candidacy prior to ECMO cannulation, which facilitates goals of care conversations. 4. Patients who are not deemed lung transplant candidates again may have their immunosuppression regimen adjusted to maximize their chances (albeit small) for recovery.

It is worth noting that the single patient in our study who received a bilateral lung transplant was on ECMO for only 3 days prior to transplant, suggesting that longer time on ECMO may be associated with higher mortality, particularly given the high rate of complications and deconditioning in this patient population that negatively impact transplantation candidacy. This is supported by our review of the literature, in which we identified an average difference of ten days (18 days vs 28 days) between ECMO initiation and lung transplant and ECMO initiation and death, respectively, in the 56 patients in whom time on ECMO was reported.

While in our center, patients on ECMO normally are required to ambulate at least 150 feet (∼45 m) to be considered transplant candidates.^44^ The International Society for Heart and Lung Transplantation (ISHLT) guidelines are less clearly defined, only going so far as to note severely limited functional status as a risk factor for substantially increased risk for poor outcome.^45^ In our series, 50% of patients died after the decision to withdraw care based on lack of transplant eligibility, with the main contraindication to transplant being the inability to meet rehabilitation goals.^46^ Even in retrospective review, the determination of why each patient was unable to meet rehabilitation goals is complex and involved a daily assessment of the patient’s clinical/hemodynamic status, the clinician’s risk assessment (as to the safety of physical activity that day), the availability of the physical therapy team, the sedation strategy, and multiple other factors. Patients despite being on V-V ECMO and invasive mechanical ventilation are frequently still quite hypoxemic, thus limiting ambulation. Due to this hypoxemia, they may require ECMO flows up to 5-7 L/min. However, achieving high flows while simultaneously awakening (i.e., minimizing/no sedation) and mobilizing patients may be limited due to drainage insufficiency (i.e., chatter, chugging, suck-down). Thus, we frequently place multiple drainage cannulas (one in each femoral vein) to allow for not only high (5-7 L/min) but also consistent ECMO flows.

Finally, many of these patients are generally healthy (median Charlson Comorbidity Index of 2 in our study) prior to hospitalization and often do well after transplant, further discussion regarding a more liberalized transplant eligibility criteria for these patients on a case-by-case basis is warranted.^42^, ^47^

Our case series faces many limitations. It is retrospective in nature, and while it is one of the larger case series to date, it is a single center experience, which limits its generalizability. The mortality rate in our series is also higher than that reported in some comparable studies. This may represent differences in illness severity or transplant eligibility criteria; however, it should be taken in consideration when deriving recommendations based on our experience. The literature review herein is limited by differences in methodology among studies, particularly by inclusion of different disease processes in addition to anti-MDA5 DM in statistical analyses. It is also limited by the lack of standardization of reported variables among studies, including variables such as sedation strategy, lung transplant approach, perioperative anti-rejection treatment protocols, days on ECMO to transplant/death, and timing of immunomodulatory treatment, all of which are essential to performing a comparative assessment to identify factors associated with successful transplant. Arguably the most significant limitation of both our case series and literature review is the presence of selection bias inherent to decisions regarding listing and delisting patients for transplant. Even at our institution, where patients are “required” to ambulate 150 ft prior to listing, the sole patient who was successfully transplanted did not fulfill that requirement. ISHLT guidelines allow for significant provider interpretation of functional status.^44^, ^45^ It is reasonable to infer that other centers (many of whose published cases are found in our literature review) have faced similar difficulty with selection bias when confronted with the uncertainty of when to list or de-list potential transplant candidates. These decisions have tremendous repercussions, as transplant offers the only reasonable hope for survival post-hospitalization, and de-listing essentially guarantees death.

In summary, early ECMO as a bridge to lung transplant for RP-ILD due to anti-MDA5 DM allows for optimization of critical aspects of pre-transplant care and should be considered for all patients when lung transplantation is a possibility. Referral to lung transplant and ECMO centers should be immediately pursued in this select group of patients. These centers should consider a less aggressive immunosuppression strategy and early transplantation to improve patient survival. Further discussion regarding transplant eligibility criteria for these patients on a case-by-case basis is recommended, and further studies are needed to identify factors associated with successful use of ECMO as a bridge to transplant in this patient population.

## Financial support

Christine M. Lin, MD, is currently supported by a grant (R21AI183310) from the National Institute of Allergy and Infectious Diseases, National Institute of Health. Mazen F. Odish, MD, is currently supported by a grant (1K23HL181397) from the National Heart, Lung, and Blood Institute, National Institute of Health.

## Patient consent

Authors confirm that appropriate patient consent to publish this case report was received.

## Conflicts of Interest statement

The authors declare that they have no known competing financial interests or personal relationships that could have appeared to influence the work reported in this paper.

## Footnotes

Appendix A Supplementary data associated with this article can be found in the online version at doi:10.1016/j.jhlto.2026.100518.
